# Women's health nurses' experiences of menopause: Considerations for nurse leaders

**DOI:** 10.1097/nmg.0000000000000023

**Published:** 2023-05-30

**Authors:** Jeanne Vanderzalm, Sadie Deschenes, Diane Kunyk

**Affiliations:** In the Faculty of Nursing at the University of Alberta, Level 3, Edmonton Clinic Health Academy in Edmonton, Alberta, Canada, **Jeanne Vanderzalm** is a retired nurse clinician and researcher; **Sadie Deschenes** is an assistant lecturer and research coordinator; and **Diane Kunyk** is a professor and acting dean.

## Abstract

This exploratory study examined nurses' experiences of menopause in relation to their caregiving abilities in an acute care setting. Menopause symptoms resulted in nurse performance issues, absenteeism, and contemplation of role changes. Interventions may help retain experienced nurses in the workforce.

**Figure:**
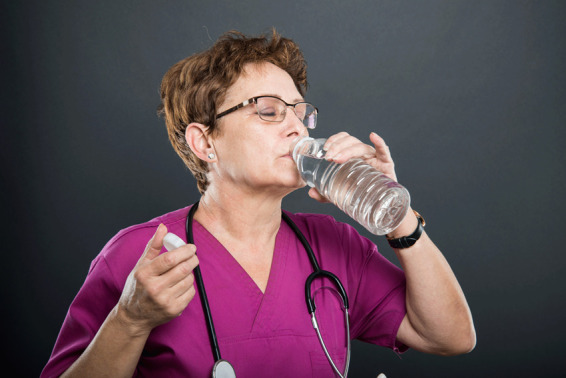
No caption available.

Within the Canadian nursing workforce, the experience of perimenopause and menopause is highly relevant as over 90% of nurses self-identify as female and 46.9% are between the ages of 35 and 54.^1^ During these ages, female nurses may experience perimenopause, menopause, and associated medical conditions.^2^,^3^ Although nurses' symptoms of perimenopause and/or menopause have been catalogued in a large, ongoing, longitudinal study of nurses, knowledge is lacking about how these symptoms may affect nursing care.^4^ This information can help nurse leaders plan for the recruitment and retention of experienced nurses and ensure the delivery of safe, quality patient care within budgetary considerations. Therefore, the purpose of this study was to examine acute care nurses' experience of menopause and perimenopause in relation to their caregiving abilities.

## Methods

Because of the lack of information about this specific area of interest, the researchers employed an exploratory qualitative method to describe the range of experiences that exist and provide a comprehensive summary of those events in everyday terminology.^5^,^6^ For this study, the researchers defined perimenopause as the period extending from the first signs of menopause to after menopause, when hormonal and biological changes occur and women may experience symptoms such as menstrual irregularities, hot flashes, night sweats, stress incontinence, insomnia, anxiety, mood changes, and decreased libido.^7^,^8^ Menopause was defined as the permanent cessation of menses for 1 year.^2^ However, as the participants in this study consistently used the word *menopause* to include perimenopausal symptoms, the researchers use the term menopause to incorporate both concepts. Prior to recruitment, the study received approval from the local health ethics research board.

The principal investigator (PI) recruited participants usingposters displayed in nurses' common areas in the hospital. Inclusion criteria were nurses who were experiencing menopause due to natural rather than surgical or medical means; self-reported menopausal symptoms; and were willing to discuss their experiences of providing patient care while experiencing menopausal symptoms. After self-selection, each nurse participated in one face-to-face audio-recorded interview of approximately 1 to 2 hours in length with the PI. Prior to each interview, the PI obtained informed consent and biographical data. During the interviews, the PI asked unstructured open-ended questions, such as “What is it like for you to be working shift work while you are menopausal?” This type of questioning enabled the participants to articulate their experiences and perceptions with minimal prompting.

Statements participants made in the first interviews guided the subsequent interviews. All interviews were audio recorded and transcribed. Two members of the research team reviewed and analyzed each transcript. They documented the themes that related across single interviews and the group as a whole. They wrote thematic memos at each analysis session to document their insights and questions. Thematic analysis continued until no new themes were identified and no new information was forthcoming from the transcriptions.

### Participants

Thirteen nurses, ages 38 to 61 years, who were providing acute care in a busy, urban women's hospital participated in this study. Situated in an older building (built in 1953), the work setting encompassed full acute care services for women (from antenatal to postpartum care), high-risk obstetrical clinics, fertility clinics, women's surgical services, gynecology, and some oncology services in an unionized environment. Seven participants self-described as direct care nurses working in varied 8- and 12-hour shifts in full-time, part-time, or casual capacity. Five cared for women in day clinics providing specialty services, and one was a nurse manager overseeing a large portfolio. Ten nurses described themselves as partnered, and eight were caring for children. Five self-described as caregiving for family members: four for aging and/or ill parents or partners and one for a child with disabilities. All participant names in this article have been changed to protect anonymity.

## Results

***Nursing during this stage of life***. The nurses in this study described physical symptoms of menopause (including sleep disturbances leading to exhaustion and fatigue) and vasomotor disturbances (including cold and hot flashes, chills, and sweats). They also reported emotional changes such as memory disruptions, anxiety, mood swings, irritability, and depression.

Fatigue resulting from sleep disturbances had workplace consequences for all participants. One 54-year-old, Paula, described her life as “navigating fatigue on a day-to-day basis. I feel the difference when I first wake up; I just don't feel rested. After I work 4 days, I am practically dragging...sometimes it takes me until the next Monday morning to sort of feel normal again.” Similarly, Mona, age 54, a clinic nurse who didn't work shifts, spoke of “unbearable hot flashes and not sleeping” and the consequences of experiencing disturbed sleep night after night. “You get up in the morning, and you're so tired that you just don't function well. Every night gets worse because you are so tired...so I get more and more panicky about it.” For Mona, fatigue leads to anxiety and questioning her own judgment: “Not sleeping, your memory kind of goes and you don't think as well, and you don't think as fast. I am sure I am not making the best judgement calls...I feel like I am losing it at times...I don't feel like I am efficient. It takes me longer to do things.”

Besides feeling like “my body doesn't want to do what I want it to do,” Dana, age 46, forces herself “to get up and cope.” She's concerned that she “can't handle the sensory overload anymore...if I have too much coming at me at the same time, I don't process that very well.” She vividly recalls a recent time at work when she felt emotionally overwhelmed and unable to cope the way she had when she wasn't menopausal:

There was one specific night shift that I was working, and it was horrendous. I was in charge and trying to handle a whole unit and, of course, the operating rooms were going, and there were no beds for patients, so patients were waiting in the hallways, and ambulances were coming. I remember saying that I can't do this anymore, and I was in tears. I had to step back and look after the light patients. I felt as if I had failed; that I just wasn't cut out for this anymore.

Phyllis, age 60, noticed that she was experiencing forgetfulness, having hot flashes at work, and feeling “constantly distracted.” and this impacted her confidence. When missing a patient's medication, she had to contact the unit to inform them, and her confidence suffered:

It jolts you and scares you when you forget something like that...because I am making my notes and I am checking my notes and I am always very concerned that I am going to miss anything. I check everything three times all of the time. Whereas I used to go around with total unconcern because I knew I had done everything. I just would do it and it was done. I never had to go back to it. But now I always worry ‘Did I 100% do that?,’ and it does stress me.

For participants, menopausal symptoms are accompanied by feelings of fear of error, loss of confidence with performing nursing skills, and frustration with their inability to cope with work. Lorraine, age 53, when speaking of work, noted “The worst thing about menopause is that feeling of not coping, just not getting done everything I want to get done.” For Phyllis, menopausal symptoms negatively affected her sense of self as a nurse: “I feel less than I was when I was young. I used to feel like I was probably very close to the best. And now I know that I am really in the crowd.” Nurses attribute these feelings to sleep deficits, vasomotor symptoms when providing nursing care, and changes with focus. As Mona noted, “I would talk to someone and couldn't even think about what they were saying. So I had to go back to ask the same questions and they would look at me like ‘is she losing it?’ And, actually, I felt like I was losing it at times.”

The nurses in this research sought solutions to mitigate the effects of their symptoms. Some attempted to “make up for the sleep disturbance by increasing the number of hours in bed,” which they found could be detrimental to partner, family, and social relationships. Another gave up, saying “I'm too tired to cope,” and made no changes. The nurses thought that if they could have restful sleep, then they'd feel less anxious, be less irritable, and could cope better with workplace stressors.

Participants varied in their desire to discuss their symptoms in the workplace. Some were open and others were very private; a few didn't acknowledge symptoms occurring when working; a number expressed embarrassment when experiencing symptoms at work; and some coped through self-deprecation. When experiencing symptoms in the workplace, some nurses felt supported by coworkers, whereas others were concerned about being considered old and unable to cope with their patient load.

***Navigating life at work***. A major concern for participants was their fatigue and its impact on their caregiving abilities. Nicole, age 44, describes making a medication error when her patient asked for Tylenol with Codeine No. 3. Nicole asked the patient about allergies and then gave her the medication. Afterward, she realized the patient had been prescribed Percocet. Nicole notes: “And that is how you make mistakes; you are so tired that it just happens.”

Nurses use various strategies to compensate for their fatigue to avoid error. Cybill, age 52, double and triple checks her work, sharing that “My husband says I say ‘I'm so tired’ in my sleep... When you are tired and worried about your concentration, you tend to check things more. I find I am always triple checking my work.” Mona also feels that her work required more time, noting, “I don't feel like I am efficient. It takes me longer to do things. I see someone and spend two hours with them, and then two weeks later, they would come in and I would ask, ‘Have I met you before?’”

Experiencing symptoms causes disruptions in workflow and fragments their focus on patient care, sometimes during procedures. For example, vasomotor disturbances distract nurses from their current tasks because they need to remove their gowns, gloves, or open doors or windows to cool themselves. Sandra, age 48, feels that she's so distracted when experiencing symptoms that she's “less patient” and “it might even affect how I start an I.V.”

Some nurses navigate life at work by removing themselves from the clinical area when they can't cope with their symptoms any longer. Leah, a 38-year-old working part-time shifts, described her experience: “I had a time when I could not sleep, and I couldn't work like that... I was too scared to make a mistake and kill somebody...that plays a role in sick time. When I answer the phone at night, and nurses are calling in sick, you often hear that they haven't slept all night.” Others have gone on medical leave to cope. Dana, age 46, who works full-time day shifts, shares that when she's having a “bad week,” she tells her physician that she “cannot deal with all of this and you have to give me the time off work. He always says, no problem, you've got it.”

Most participants were seeking out role changes, hoping it would mitigate their symptoms. Some nurses decrease their full-time to part-time hours; others look for positions that don't require shift work. One nurse sacrificed her practice experience, clinical skills, and personal interests to pursue an undesired management position. Although they often spoke about it, none had made concrete plans to leave the profession. As experienced nurses, they had either invested many years (but often not enough) in pension accrual, or their personal responsibilities wouldn't allow them to retire. They felt that their only option was to continue to work despite experiencing physical symptoms that they worried would compromise their ability to perform care safely.

***Workplace adaptations***. Participants felt that workplace adaptations would be a benefit when navigating menopausal symptoms and improve their patients' care experiences. Most worked in areas that lacked windows, had poor ventilation, and were just “too hot.” One participant wished she could lower the room temperature because it was “pretty hard to concentrate while having to mop the sweat off your face,” and another notes being “very conscious not to lean over patients in case I drop [sweat] on them.” Mona articulated the impact on her patients when having a “hot flash” during a procedure:

You cannot just leave... I am taking my lab coat off, my gloves off, opening the door, and trying to get some air. So, it is taking longer to do my treatment. It's disruptive to them too because they wonder why I am opening the door...do I [the patient] smell? But it's just the hot flashes.

When probed about recommendations, Mona suggested: “I would make the rooms a little bigger, I would certainly make the air conditioning work or have windows available that could open.” She noted that natural light would be beneficial because “if we turn the lights on, we get so hot that no one can work there.” To help nurses cope, Phyllis advocated for “a more comfortable lounge area, having a consistent temperature with muted lighting, and chairs that lay back for napping” and further stressed the importance of “access to proper food when you have to work overtime.” However, the nurses in this study weren't confident that any changes in their workplace, which they viewed as “inflexible,” would be possible. Leah, when speaking of “noon hour yoga,” noted that its timing was incongruent with hospital routines. “It seems like the environment is not conducive to people being healthy; even things that are set up for hospital employees really aren't.”

Nurse participants stressed the need for more flexibility with their workloads and time management. One frequently desired option was to “change patient loads if experiencing severe symptoms that day.” In their opinions, self-shift scheduling or adjusting work hours would reduce the need for sick leave. Rather than call in sick, Charlotte, age 51, said: “It would be nice to phone and say I didn't sleep well last night so I will start a little later coming in.” Saleema, driven by fatigue to the point of considering leaving her position, wondered whether employers would say “we can hire you back by contract...there are options for us to keep you.” Paula emphasized that “at the very least, we should not be expected to work 12-hour shifts anymore.” Overall, the nurses in this study concluded that their performance, and ultimately patient care, would benefit from workplace adaptations for their vasomotor and sleep disturbances.

## Discussion

This study explored 13 acute care women's health nurses' experiences of menopause and its related impact on their provision of care. Participants reported experiencing extreme fatigue resulting from sleep disturbances, anxiety regarding the safety of their care provision, and concern for their patients' care experiences. Their quandary between wanting to continue working and their experiences of fatigue, memory loss, and anxiety led most to explore other nursing roles in order to cope.

Nurse participants in this study said they didn't discuss their menopausal symptoms with their manager for fear of being seen as “old and unable to cope,” which was consistent with the findings of a previous study by Norton and Tremayne.^9^ This finding reinforces the importance of nurse leaders being educated about menopausal symptoms and changes, exhibiting an awareness of and sensitivity to ageism, and focusing resources on improving the climate of the working environment.^3^ Leaders need to be knowledgeable, proactive, and sensitive about the symptoms nurses may be experiencing, and pay far more attention to menopause as a workplace issue for pressing social responsibility, demographic, legal, and business reasons.^10^,^11^

Menopause among female nursing staff is a worthy consideration for nurse leaders concerned with patient safety, human resources costs, and retention of their experienced nursing workforce. The nurses in this study draw attention to the risks associated with extreme fatigue, such as errors and inefficiencies, and a connection to absenteeism, medical leaves, and contemplation of role changes or leaving the profession. It's concerning that nurses may leave their current and preferred area of work despite having the skills, experience, and passion for the profession simply to seek employment that provides adaptations for this biological change with hope that their risks of making an error will be reduced or eliminated.

The nurses in this study made recommendations regarding workplace changes, such as controlling temperature, places to rest, and access to nutritious food and water. Oftentimes, nurses described the immediacy of needing relief from vasomotor disturbances that disrupted patient procedures. The increased use of personal protective equipment such as face shields during the current global pandemic can exacerbate these symptoms, heightening this concern for nurses experiencing menopause. The ability to control temperature and ventilation in the workplace environment is an established standard recommendation for menopausal individuals.^12^ The nurses in this study frequently raised scheduling as an issue, suggesting self-scheduling and part-time positions for flexibility, recommendations that have been previously identified in the literature.^12^ Environmental changes to reduce vasomotor disturbances, adjusting shifts to lessen fatigue, and the creation of a culture of acceptance and sensitivity to ageism are important steps for consideration.

## Implications for nurse leaders

This study describes the workplace experience of acute care nurses having menopausal symptoms. Menopause is a worthy consideration for nurse leaders concerned with patient safety and retaining their experienced nursing workforce, given that over 90% of nurses are female, and almost half of those are of menopausal age. The fact that the nurses in this study reported that their extreme fatigue resulted in performance issues, such as errors and inefficiencies, absenteeism, medical leaves, and the contemplation of role changes or exiting the profession should be of particular concern to nurse leaders.

It's important to consider these dangerous, costly, and destabilizing outcomes during times of nursing shortages. It would be more productive to implement interventions to make the workplace amenable for nurses experiencing menopause, such as packaging items for procedures, providing checklists to decrease errors, or instituting methods to ensure double-checking of nursing-care practices. Introducing wellness programs in the workplace to promote lifestyle modifications, exercise, sleep hygiene, inclusivity, and acceptance of different nurses' experiences could better support nurses during menopause. Additionally, developing policies that encourage employees to take vacation time, allow for workplace changes (such as controlling the temperature, ensuring water is available, and having designated places to rest), and heightened awareness of menopausal symptoms among management could further allow management to meet the needs of their aging workforce.

As advocates for their workforce, nurse leaders must acknowledge that nurses may experience difficulties during this stage of life. Offering support developing alternate work plans and making adjustments to working conditions for nurses experiencing difficulties will not only enhance nurses' work life but will help retain experienced clinical nurses in the workforce.

## Research criteria

**Purpose:** To examine nurses' experiences of menopause in relation to their caregiving abilities in an acute care setting.

**Background:** Menopause is an important consideration for nurse leaders because the nursing workforce remains predominantly female. However, knowledge is lacking about the manner in which menopausal symptoms may affect nursing care in acute care settings.

**Methods:** An exploratory qualitative method was employed to describe how nurses' menopausal experiences impacted their nursing practice. Researchers interviewed 13 nurses, ages 38 to 61, from an urban women's hospital.

**Results:** Nurses describe experiencing extreme fatigue, memory lapses, workplace inefficiencies, lack of confidence and coping abilities, anxiety regarding the safety of their nursing care, and concern for patient's care experiences. To cope, they consider resigning, position changes, reduced hours, and medical leaves.

**Conclusion:** In this exploratory study, symptoms of menopause resulted in nurse performance issues, absenteeism, and contemplation of role changes. Workplace adaptations may encourage the retention of nurses at the peak of their productive lives and positively impact the nursing shortage, quality care delivery, and operational budgets.

**Nursing implications:** Interventions to improve working conditions and support the navigation of menopausal challenges may enhance nurses' work lives and retain experienced nurses in the workforce. Further study is required to determine the impact of the recommendations and their generalizability to other practice settings.
